# Comparison between the Transcutaneous and Total Serum Bilirubin Measurement in Malay Neonates with Neonatal Jaundice

**DOI:** 10.21315/mjms2022.29.1.5

**Published:** 2022-02-23

**Authors:** Mazrah Mohamed, Nor Rosidah Ibrahim, Noraida Ramli, Noorizan Abdul Majid, Najib Majdi Yacob, Ariffin Nasir

**Affiliations:** 1 Department of Paediatrics, School of Medical Sciences, Universiti Sains Malaysia, Kubang Kerian, Kelantan, Malaysia; 2 Hospital Universiti Sains Malaysia, Universiti Sains Malaysia, Kubang Kerian, Kelantan, Malaysia; 3 Biostatistic and Research Methodology Unit, School of Medical Sciences, Universiti Sains Malaysia, Kubang Kerian, Kelantan, Malaysia

**Keywords:** transcutaneous bilirubin, total serum bilirubin, JM-105 bilirubinometer, neonatal jaundice

## Abstract

**Background:**

This study aims to investigate the reliability of the Dräger Jaundice Meter JM-105 for the screening of neonatal jaundice in Malay neonates.

**Methods:**

A cross-sectional study was conducted in a university hospital involving 130 jaundiced neonates requiring serum bilirubin determination from day 2 to day 7 of life.

**Results:**

The mean total serum bilirubin (TSB) was 232 μmol/L, whereas the mean transcutaneous bilirubin (TcB) measured at the forehead and sternum were 222 μmol/L and 223 μmol/L, respectively. Further, TcB underestimates TSB with a mean difference of 10.10 μmol/L at the forehead and 9.27 μmol/L at the sternum. A positive linear relationship was observed between TSB with TcB forehead (*r* = 0.82) and TcB sternum (*r* = 0.80). A good discriminations ability was observed for both the TcB forehead (receiver operating characteristics [ROC] curve = 89.8%) and sternum (ROC curve = 89.7%) at a TSB level of 205 μmol/L. The sensitivity ranges from 84.4% to 85.3%, while the specificity ranges from 77.4% to 76.4%.

**Conclusion:**

Our study demonstrates a strong linear relationship and good diagnostic accuracy of TcB values compared to TSB values. To conclude, TcB measured at the forehead or sternum is a good alternative as a non-invasive screening tool for non-severe hyperbilirubinemia in Malay neonates.

## Introduction

Neonatal jaundice is one of the commonest causes of hospital admission during the first week after birth. It is occurs due to hyperbilirubinemia (1, 2). Most cases are benign, but severe neonatal hyperbilirubinemia can lead to bilirubin encephalopathy (kernicterus). Kernicterus is associated with a high mortality rate and survivors usually suffer from complications such as athetoid cerebral palsy, high-frequency hearing loss and intellectual disability (3). Severe neonatal hyperbilirubinemia and its sequelae can be prevented with appropriate serum bilirubin monitoring and early treatment involving phototherapy or exchange blood transfusion.

To measure bilirubin levels, the total serum bilirubin (TSB) measured by the biochemical laboratory is still considered a gold standard, but it is invasive, requiring needle pricks that carry the risk for infection, and cause pain and stress to the neonates (4). The turnaround time for bilirubin test results may delay the initiation of therapy for neonatal hyperbilirubinemia. In recent years, the transcutaneous bilirubinometer, which uses photometry to detect bilirubin levels, has been used as an alternative to estimate the bilirubin levels.

A transcutaneous bilirubinometer is a portable, painless and non-invasive device. The bilirubin is estimated by pressing a probe either to the neonatal forehead or sternum. It gives an immediate result, allowing the immediate initiation of therapy and reducing the burden borne by health care providers.

The first transcutaneous bilirubinometer was introduced in the 1980s and the technologies related to the devices have evolved tremendously over the past few decades. Since then, numerous studies using various transcutaneous bilirubinometer devices have been conducted to prove its accuracy and sensitivity. The good correlation between TSB and transcutaneous bilirubin (TcB) values has made the transcutaneous bilirubinometer a valuable screening tool for TSB in hyperbilirubinemia management worldwide (7, 8). The American Academy of Paediatrics even recommends a pre-discharge evaluation of bilirubin levels by measuring TSB or TcB in all neonates (8). Some countries as India and Mongolia had, in fact, conducted studies to implement the transcutaneous bilirubinometer as the screening tool for neonatal jaundice in their rural populations as well as those lacking resources (9, 10).

However, there were only limited studies done using transcutaneous bilirubinometer in our country (11). Since different levels of skin pigmentation may theoretically influence results, this study aims to investigate the reliability of a transcutaneous bilirubinometer (the Dräger Jaundice Meter JM-105) for the screening of neonatal jaundice in a predominantly Malay population.

## Methods

This was a cross-sectional study conducted in a tertiary university hospital, the largest hospital in the northeastern part of Malaysia. The study took place in a special care nursery for two months in 2018.

Eligible neonates were clinically jaundiced and required serum bilirubin determination. They were born at more than 35 weeks of gestation, had a birth weight of more than 2 kg, and were aged between 2 days and 7 days. Exclusion criteria were severely ill neonates, neonates with a lethal congenital malformation, and those who had neonatal jaundice, and had already been treated with phototherapy. The written informed consent was obtained from parents or legal guardians of the neonates, involved in the study. Their routine TSB was obtained as part of the standard practice in the unit. The serum bilirubin samples were sent to the biochemical laboratory within one hour of blood taking. The TSB samples were processed at the hospital biochemical laboratory by the colorimetric method using the Architect C800 and Olympus AU400 analyser.

Following this, TcB measurements were taken by the researcher or a trained research assistant at the neonates’ forehead and sternum site, using the Dräger Jaundice Meter JM-105 within one hour of the serum bilirubin sampling. At each site, three measurements of TcB were taken, and the mean of these values was recorded as the mean TcB. The doctor in charge decided the therapy based on the serum bilirubin level.

## Statistical Methods

Data entry and analysis were performed using the IBM SPSS software version 26. Demographic data among the neonates were grouped and analysed using descriptive analysis. Normally distributed numerical data were presented as mean and standard deviation (SD), while categorical data were presented as frequency (*n*) and percentage (%). The mean difference between TSB and mean TcB measured by the Dräger Jaundice Meter JM-105 at the forehead and sternum were determined using a one-way repeated-measures analysis of variance (RM ANOVA).

Further statistical analysis was conducted using the STATA software version 14. Linear regression by the method of ordinary least squares (OLS) was used to assess the strength of the linear relationship and the systematic error (bias) of the forehead TcB and sternum TcB compared to the TSB. The general relationship between methods was shown by the angle of the line (the slope) and its interception with the *y*-axis. The direction and strength of linear relationship were shown by the correlation coefficient (*r*) and the coefficient of determination (*R*^2^). The Bland-Altman plots were evaluated for assessment of bias across the measurement range. The discriminant ability of the forehead TcB and sternum TcB to discriminate TSB at a cut-off point of 205 mmol/L were evaluated by assessing the area under the receiver operating characteristics (ROC) curve. A higher ROC curve indicates a better discriminant ability of the measurement method. Sensitivity, specificity, positive and negative predictive values of the forehead TcB and sternum TcB were calculated using a similar cut-off point of TSB (205 mmol/L). To identify the difference between the TcB and TSB values at various TSB levels, the difference (TcB minus TSB) values were plotted against the TSB values. Values of more than 0 indicated that the TcB values were larger than the TSB values (TcB overestimate TSB), and differences of less than 0 indicated that the TcB values were lower than the TSB (TcB underestimate TSB).

## Results

**Figure 1:**
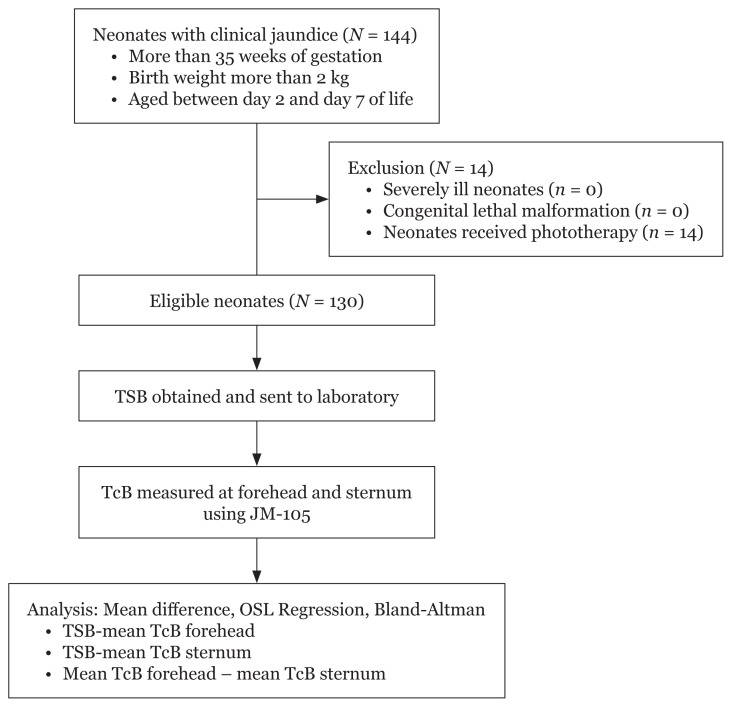
Study flow chart

There were 144 neonates with clinical jaundice who fulfilled the inclusion criteria during the study period (Figure 1). Fourteen of them were excluded from the study as they had received phototherapy. Therefore, 130 neonates were recruited for this study. The demographic characteristics of the neonates involved in the study are shown in Table 1. All were Malay neonates. To elaborate, 66 neonates (51%) involved had been borne more than three days ago. More than half were female (56.2%). The mean birth weight was 3.02 (0.46) kg, ranging from 2.05 kg to 4.40 kg. The mean gestational age was 38.4 (1.31) weeks, ranging from 35 weeks to 42 weeks.

Jaundiced term neonates (78.5%) outnumbered jaundiced late preterm neonates (21.5%). Most neonates were born via spontaneous vaginal deliveries (76.2%). Mothers with an O positive blood group (46.1%) were the most frequent in this study, while the least frequent were mothers with an AB positive blood group (6.2%). Most neonates in this study had an O positive blood group (40%) followed by neonates with a B positive blood group (29.2%). More than half of the neonates in this study had a positive family history of neonatal jaundice (55.4%). About half of the neonates were exclusively breastfed, while the others had mixed feeding (breast feeding topped with formula).

The mean TSB level was 232 (72) μmol/L, with values ranging from 74 μmol/L to 419 μmol/L. Further, nine neonates (6.9%) had TSB ranged between 340 μmol/L and 419 μmol/L, and were treated with intensive phototherapy, according to the neonatal management protocol. The mean TcB level measured at the forehead was 222 (55) μmol/L, with values ranging from 93 μmol/L to 349 μmol/L. The mean TcB level measured at the sternum was 223 (59) μmol/L, with values ranging from 73 μmol/L to 346 μmol/L.

The one-way RM ANOVA for comparison of the forehead TcB, sternum TcB and TSB indicated that the sphericity assumption was violated: Mauchly’s W (df = 2) = 0.69, *P* < 0.001. The Greenhouse-Geisser epsilon correction was used to evaluate the test of within-subjects’ effects. The overall *F*-test with the Greenhouse-Geisser method was significant, *F* (1.53, 197.36), *P* = 0.007. A pairwise comparison with the Bonferroni’s adjustment method for multiple comparisons was made, and the results are summarised in Table 2. The results indicate that TSB’s mean was significantly higher than the mean of TcB measured at the forehead (*P* = 0.019) and sternum (*P* = 0.048).

**Figure 2:**
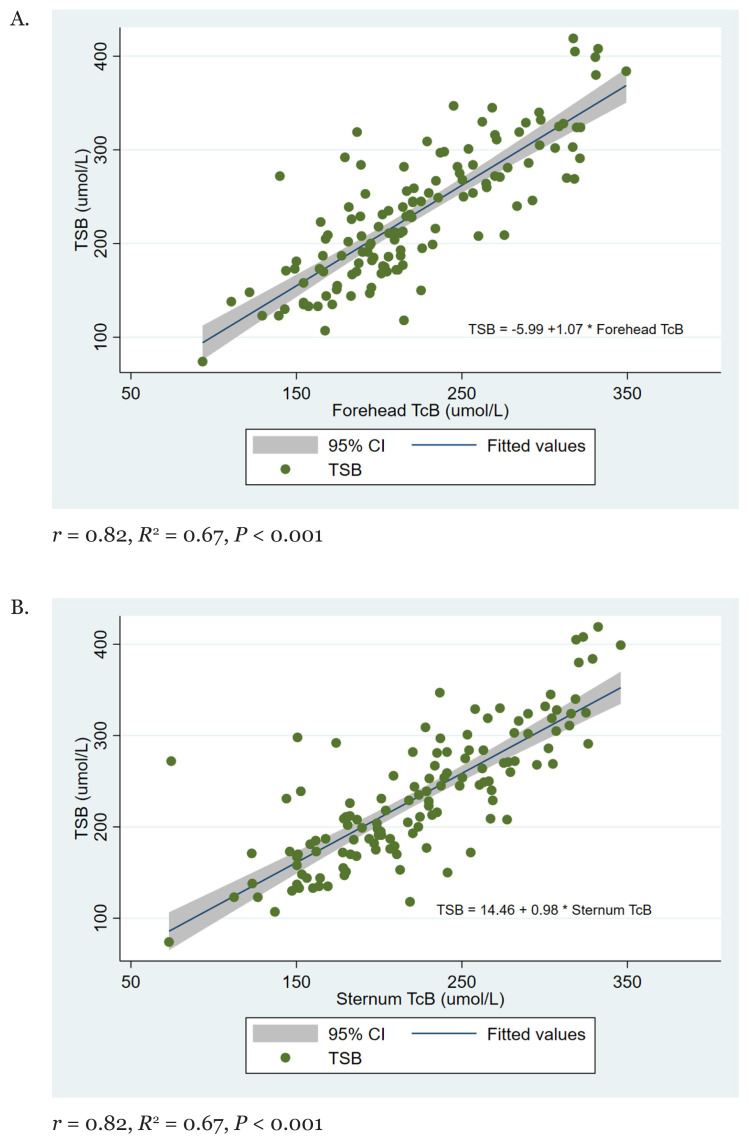
Linear regression plot for (A) TSB versus the forehead TcB, and (B) TSB versus the sternum TcB

OLS linear regression analysis for TSB and forehead TcB indicates a significant positive strong linear relationship between the two measurements (*r* = 0.82, *R*^2^ = 0.67, *P* < 0.001) (Figure 2a). A similar pattern of linear relationship was observed between the sternum TcB and TSB (*r* = 0.80, *R*^2^ = 0.64, *P* < 0.001) (Figure 2b).

**Figure 3:**
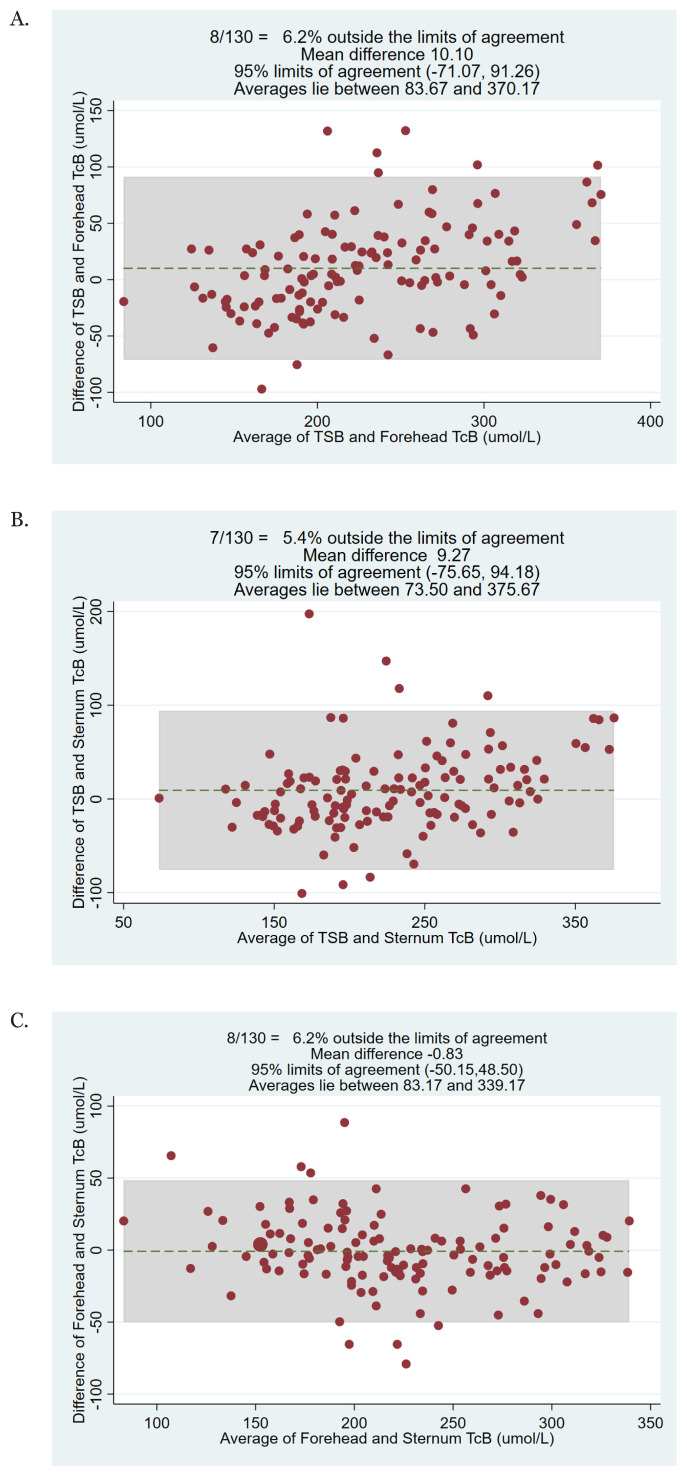
Bland-Altman plot showing the difference and average of (A) the forehead TcB and sternum TcB, (B) sternum TcB and TSB, and (C) forehead TcB and sternum TcB

The Bland-Altman plot for TSB versus the forehead TcB showed good agreement between the tests, with only 8/130 (6.2%) of the tests being located outside the limits of agreement (Figure 3A). A similar pattern was observed for the comparison between TSB versus the sternum TcB (only 7/130 [5.4%] were outside the limits of agreement) (Figure 3b) and between the forehead TcB versus the sternum TcB (only 8/130 (6.2%) of the tests that were outside the limits of agreement) (Figure 3C).

**Figure 4:**
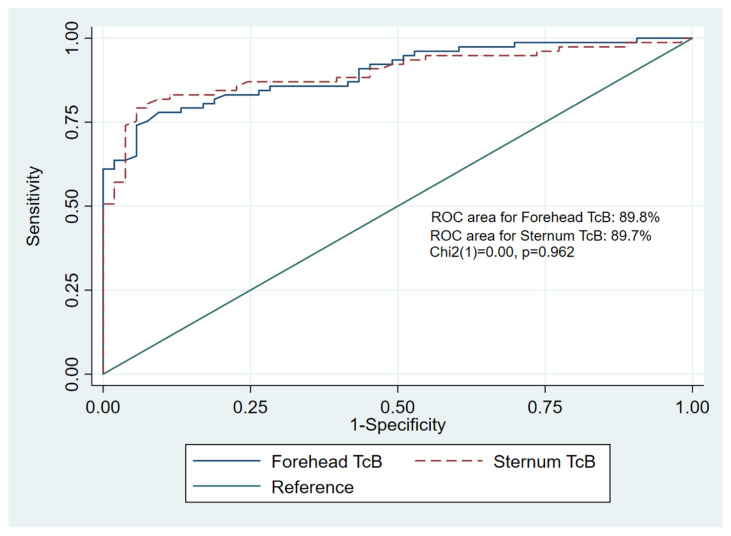
Comparison of the area under receiver operating characteristics (ROC) curve between the forehead TcB and sternum TcB at TSB level of 205 μmol/L

Using a common cut-off for phototherapy according to the Malaysian Ministry of Health Clinical Practice Guidelines for the management of neonatal jaundice (bilirubin level of 205 mmol/L), the area under the ROC curve was 89.8% for forehead TcB and 89.7% for sternum TcB. The statistical comparison of the two ROC curves indicates no significant difference, c^2^ (1) = 0.00, *P* = 0.962 (Figure 4). The diagnostic accuracy of the TcB measured at the forehead and sternum at a TSB level of 205 mmol/L indicates that there are not much differences in term of sensitivity, specificity, positive as well as negative predictive values (Table 3).

**Figure 5:**
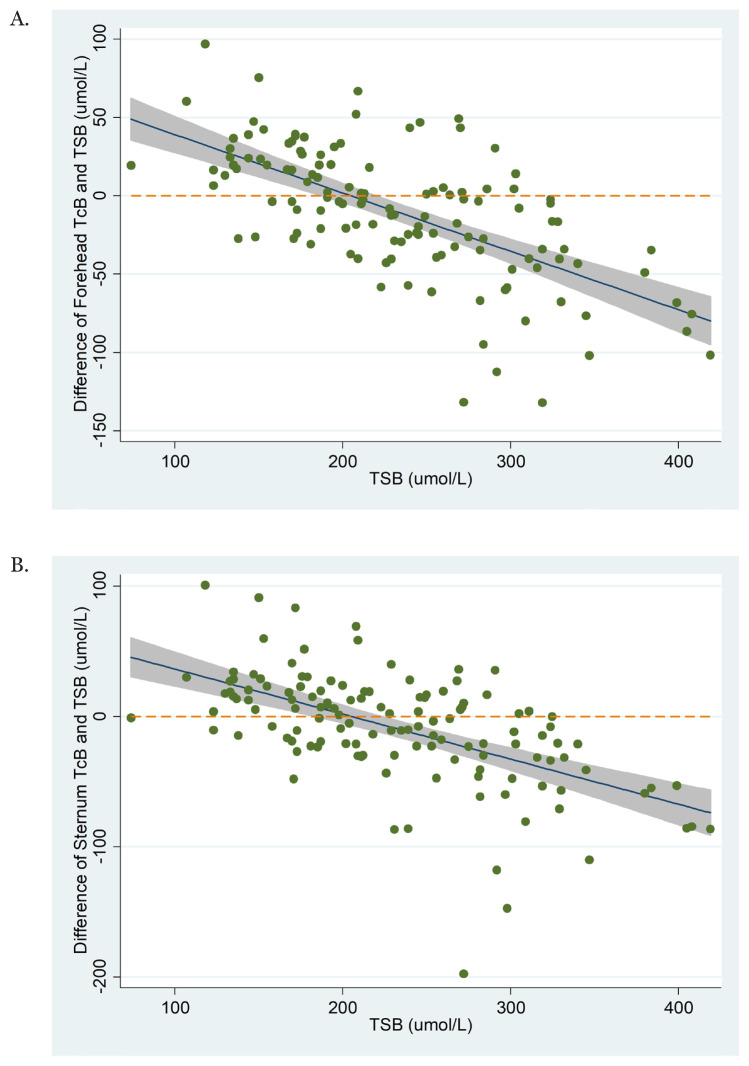
Characterisation of the difference between (A) the forehead TcB-TSB and (B) sternum TcB-TSB throughout the measurement range of TSB. The shaded area represents the 95% CI of the linear fit line

Figure 5 illustrates the difference between TcB and TSB values throughout the measurement range of TSB. For both TcB measurements (at the forehead and the sternum), the fit line indicates that at lower TSB values, TcB overestimates TSB. At around TSB values of 200 mmol/L, TcB measurements started to underestimate the TSB values. The underestimation of TSB values by TcB is gradually greater as the TSB values increase.

## Discussion

This is the first study using a transcutaneous bilirubinometer focusing on the Malay ethnicity. However, a previous study in our country by Boo and Ishak (11) also showed nearly similar results with our study. The minor difference between our study and the study mentioned earlier could be attributed to the larger number of participants involved in their study (*n* = 345) with a diversity of races involved (Malays, Chinese and Indians) and a different type of bilirubinometer (Bilicheck) used.

Our results showed that at a lower TSB value (below 200 mmol/L), TcB tends to overestimate the TSB. However, starting from TSB 200 mmol/L onward, TcB values underestimate the TSB values. Our study demonstrated similar results with other studies (13–15) using different types of bilirubinometers.

Rodríguez-Capote et al. (13) using two types of bilirubinometers (BiliCheck and JM-103), conducted a study in Canada involving neonates of more than 35 weeks of gestational age, with half of the neonates being Caucasian. They found a good correlation between TSB and TcB estimation from both devices, with TcB underestimated the TSB values. A study done by Engle et al. (14) with Hispanic neonates was the predominant ethnic group. Using a BiliCheck bilirubinometer involving 304 late preterms and term neonates showed that despite good correlation (*r* = 0.84), the transcutaneous bilirubinometer reading tended to underestimate the TSB value in the higher serum bilirubin level. Similarly, Şimşek et al., who studied 250 Turkish neonates of more than 36 weeks of gestational age, also reported underestimating TSB values using the transcutaneous bilirubinometer device in their study (15). The similarity of the TcB in our study and the studies mentioned earlier might be possible due to the similarity in the gestational age of neonates enrolled in the study. We also suggested it might be possible because those neonates had similar skin tone, leading to the similarity in underestimation of the TcB.

The difference in the TSB estimation values by our study and the previous studies may be contributed by racial variation and differences in skin tones. TcB levels in the races with darker skin colour such as Indian and black Africans tend to be overestimated compared to TSB. Studies by Jandial et al. (16) in India and Olusanya et al. (17) in Africa also observed a TcB overestimation compared to the TSB, using Drager JM-103 in India and BiliCheck and JM-103 in black Africans neonates. This overestimation may result in unnecessary phototherapy treatment for neonatal jaundice.

This study found a strong linear relationship between TSB with both the mean TcB forehead (*r* = 0.82) and mean TcB sternum (*r* = 0.80). This observation is similar to a study by Taylor et al. (4) where data were obtained from 27 nursery sites involving 925 matched TcB and TSB level from both the chest and forehead. They used two brands of the TcB device (BiliCheck and JM-103) and found a good correlation between TSB and TcB values. In Iran, Mansouri et al. (6), with a wider range of neonatal ages, between 1 day and 22 days, also found a good correlation coefficient between 200 neonates, TSB and TcB forehead. Our results were similar to other studies in other Asian countries, such as those conducted in Thailand, India and Hongkong (18–20).

Rubaltelli et al. (21) reported a good positive linear relationship between TSB and TcB, with the TcB forehead values (*r* = 0.89) being better than the TcB sternal values (*r* = 0.88). However, other studies found different results (22–24). Kosarat and Khuwuthyakorn’s (22) study in 2016, involving 257 neonates, found that the TcB sternal measurement had a better correlation coefficient (*r* > 0.8) with TSB than the TcB forehead measurement. Chimhini et al. also revealed a similar finding (23). This study conducted in Zimbabwean neonates reported that the sternum was better at evaluating neonates with jaundice than the forehead, with the correlation between the TSB and TcB sternum being 0.77 and between TSB and TcB forehead being 0.72. Another study conducted in Egypt by El-Kabbany et al. also showed a significant correlation between the TSB and TcB forehead (*P*-value < 0.05) and between TSB and TcB sternum (*P*-value < 0.001) with sternal measurements being more accurate than forehead measurements (24).

We concluded that the observed differences in the TcB measured at the forehead and sternum might be due to the difference in exposure of light on the skin. The chest area is more ‘light protected’ by clothes, while the forehead is more exposed to the light. Also, the skin composition variation as collagen and melanin concentration may affect the wavelength detected by the TcB photometry, causing this difference in TcB estimation. Furthermore, in neonates, the hair is less thick in the chest area than the forehead, contributing to this difference (11).

Moreover, nine infants (6.9%) had severe hyperbilirubinemia ranging from 340 mmol/L to 419 mmol/L in our study. However, all of them was resolved with intensive phototherapy. Despite high TSB values, we found that the device can only display an estimation value of up to 340 mmol/L. TcB results above 340 will result in the device not revealing the exact TcB value. The measurement limit was specifically mentioned in the manufacturer guideline (the maximum level of TcB detection is 340 mmol/L).

In our study, most TcB value initially underestimated TSB value until a certain cut-off point. The following is an especially important limitation that one has to be aware of: once TSB is more than 340 mmol/L, the TcB becomes unreliable in estimating the TSB value. The misinterpretation of the results may cause a significant difference in the management of infants with severe jaundice. The undertreatment of this group may lead to complications such as kernicterus and its comorbidities, while on the other hand, overtreatment may cause redundant blood taking and possible unnecessary exchange transfusion. Thus, we can conclude that the transcutaneous device should be used with precaution, especially when interpreting the high TcB level. In high levels of TcB, TSB should be used to confirm the bilirubin level. Further management of neonatal jaundice as phototherapy or exchange transfusion should be guided by the serum bilirubin level.

Our study was a single-setting one with a relatively small sample size when compared to other published studies. The neonates were only confined to a small population in one hospital setting; we did not conduct the study in a larger population involving secondary and primary health. The study was conducted in Hospital Universiti Sains Malaysia, where most of the patients were Malays who lack ethnic diversity. Therefore, this study may not be applicable to other major Malaysian ethnic groups, i.e. Chinese and Indians. It is essential to have more studies involving more races and skin tone as the later may affect the performance of the transcutaneous bilirubinometer (23).

Our study only involved healthy late preterm or term neonates before their treatment with phototherapy. Further studies should be conducted to evaluate the TcB’s relation to TSB in smaller premature neonates or high-risk and ill neonates. The pre- and post-phototherapy TcB relation to TSB should also be studied to better evaluate the usefulness of the TcB device in our population. Another step to explore is evaluating an hour-specific TcB nomogram in our population. Throughout the years, TcB values were plotted against the Bhutani TSB nomogram for the initiation of phototherapy in neonates (25). Mohamed et al. (26) suggested plotting the TCB values against Maisel’s nomogram. Further studies should be conducted to aid in developing a population-specific TcB nomogram for a more accurate prediction of hyperbilirubinemia in our community.

## Conclusion

The study demonstrated a strong linear relationship between TcB values and TSB values. Measurements of TcB at the forehead and sternum showed good diagnostic accuracy for discriminating neonates requiring phototherapy. We also found that the TcB value may underestimate or overestimate the TSB value of about 50 mmol/L. This is especially important in managing severe hyperbilirubinemia in neonates with a TSB that is more than 340 mmol/L, where the TcB estimation may unreliably estimate the TSB. Even though using a bilirubinometer for the TcB measurement at the forehead or sternum is a good convenient alternative as a non-invasive screening tool for non-severe hyperbilirubinemia in Malay neonates, one should be careful and it would be advisable to use the TSB instead of solely depending on TcB alone when it comes to patients with severe hyperbilirubinaemia.

## Figures and Tables

**Table 1:** Demographic characteristics (*N* = 130)

	*n* (%)
Gender	
Female	73 (56.2)
Male	57 (43.8)
Birth weight (g)	
2,000–2,500	17 (13.1)
≥ 2,501	113 (86.9)
Gestational age (weeks)	
35–36	28 (21.5)
37–42	102 (78.5)
G6PD status	
Deficient	9 (6.9)
Normal	121 (93.1)
Mother’s blood group	
A positive	30 (23.1)
B positive	32 (24.6)
O positive	60 (46.1)
AB positive	8 (6.2)
Baby’s blood group	
A positive	34 (26.2)
B positive	38 (29.2)
O positive	52 (40)
AB positive	6 (4.6)
Mode of delivery	
LSCS	31 (23.8)
SVD	99 (76.2)
Family history with neonatal jaundice	
Yes	72 (55.4)
No	58 (44.6)
Feeding	
Exclusive breast feeding	66 (50.8)
Mixed feeding	64 (49.2)

Notes*:* LSCS = lower segment caesarean section; SVD = spontaneous vaginal delivery

**Table 2:** Multiple pairwise comparisons of TSB, sternum and forehead TcB

Comparison	Mean difference	95% CI of difference	*P*-value*
TSB versus forehead TcB	10.10	1.29, 18.90	0.019
TSB versus sternum TcB	9.27	0.05, 18.48	0.048
Forehead TcB versus sternum TcB	−0.83	−6.18, 4.53	> 0.95

Note:

* Adjustment for multiple comparisons using the Bonferroni method

**Table 3:** Diagnostic accuracy of the forehead TcB and sternum TcB compared to the TSB at the cut-off level TSB > 205 μmol/L

	Forehead TcB (%)		Sternum TcB (%)	
	
	Estimate	95% CI	Estimate	95% CI
Sensitivity	85.3	79.3, 91.4	84.4	78.2, 90.7
Specificity	76.4	69.1, 83.7	77.4	70.2, 84.6
Positive predictive value	83.1	76.7, 89.6	84.4	78.2, 90.7
Negative predictive value	79.3	72.3, 86.2	77.4	70.2, 84.6
